# Prevalence of obesity and abdominal obesity in the Lausanne population

**DOI:** 10.1186/1471-2458-8-330

**Published:** 2008-09-24

**Authors:** Pedro Marques-Vidal, Murielle Bochud, Vincent Mooser, Fred Paccaud, Gérard Waeber, Peter Vollenweider

**Affiliations:** 1 Cardiomet, CHUV, Lausanne, Switzerland; 2 Institute of Social and Preventive Medicine (IUMSP), University of Lausanne, Switzerland; 3 Genetics Division, GlaxoSmithKline, Philadelphia, Pennsylvania, USA; 4 Department of Medicine, Internal Medicine, CHUV, Lausanne, Switzerland

## Abstract

**Background:**

Obesity can be defined using body mass index (BMI) or waist (abdominal obesity). Little information exists regarding its prevalence and determinants in Switzerland. Hence, we assessed the levels of obesity as defined by BMI or waist circumference in a Swiss population-based sample.

**Methods:**

Cross-sectional, population-based non-stratified random sample of 3,249 women and 2,937 men aged 35–75 years living in Lausanne, Switzerland. Overall participation rate was 41%.

**Results:**

In men, the prevalences of overweight (BMI ≥25 kg/m^2^) and obesity (BMI ≥30 kg/m^2^) were 45.5% and 16.9%, respectively, higher than in women (28.3% and 14.3%, respectively). The prevalence of abdominal obesity (waist ≥102 in men and ≥88 cm in women) was higher in women than in men (30.6% vs. 23.9%). Obesity and abdominal obesity increased with age and decreased with higher educational level in both genders. In women, the prevalence of obesity was lower among former and current smokers, whereas in men the prevalence of obesity was higher in former smokers but did not differ between current and never smokers. Multivariate analysis showed age to be positively related, and education and physical activity to be negatively related with obesity and abdominal obesity in both genders, whereas differential effects of smoking were found between genders.

**Conclusion:**

The prevalence of abdominal obesity is higher than BMI-derived obesity in the Swiss population. Women presented with more abdominal obesity than men. The association between smoking and obesity levels appears to differ between genders.

## Background

For almost 250 years, obesity has been defined as an increased body fat [1], but as body fat has rarely been directly assessed, surrogate variables such as body mass index (BMI) or waist have been used. The most currently used definition of obesity is based on BMI [1], although another definition, based on waist circumference (i.e. abdominal obesity), is also frequently used [1]. Several studies have shown that waist is more closely associated to all-cause mortality or cardiovascular risk factors than BMI [2-5] and that, in subjects with normal BMI, increased waist is associated with higher levels of cardiovascular risk factors [6,7]. There is little evidence whether using different anthropometric indices can lead to similar estimations of the prevalence of obesity in the general population [8,9], and recent information on the prevalence and determinants of obesity and increased waist (abdominal obesity) in the Swiss population are scarce.

In this study, we used data from a large, population-based examination survey conducted in Lausanne to assess the prevalence of obesity as measured by BMI and waist levels.

## Methods

The recruitment process of the CoLaus study has been described elsewhere [10]. Briefly, the complete list of the Lausanne inhabitants aged 35–75 years (n = 56,694) was provided by the population registry of the city. A simple, non-stratified random selection of the subjects was performed and a random sample of 35% of the overall population was drawn. An invitation letter with a quick description of the study and a formulary in a pre-stamped envelope was sent to all randomized subjects. Subjects interested in participating returned the formulary and were contacted telephonically within 14 days by one of the staff members who provided more information about the study and arranged for an appointment. The study was approved by the Ethics Committee of the University of Lausanne and written informed consent was obtained from participants before data collection.

Data on smoking, education (basic, apprenticeship, high school and university) and physical activity (none vs at least once/week) were collected by trained field interviewers. Body weight and height were measured with participants standing without shoes in light indoor clothes. Body weight was measured in kilograms to the nearest 100 g using a Seca^® ^scale, which was calibrated regularly. Height was measured to the nearest 5 mm using a Seca^® ^height gauge. Obesity was defined as BMI ≥ 30 kg/m^2 ^[1].

Waist was measured with a non-stretchable tape over the unclothed abdomen at the narrowest point between the lowest rib and the iliac crest [11]. Hip was measured as recommended using a similar procedure [11]. Two measures were made and the mean (expressed in centimeters) used for analyses. Obesity was defined as waist ≥ 102 cm for men and ≥ 88 cm for women [1,11].

Statistical analysis was conducted using Stata 9.2 for Windows (Stata Corp LP, Texas, USA). Comparisons were conducted using chi-square or logistic regression. Statistical significance was established for p < 0.05.

## Results

We assessed 3,249 women and 2,937 men (53.1 ± 10.8 years, mean ± SD). Men had higher BMI: 26.6 ± 4.0 (mean ± standard deviation) *vs*. 25.1 ± 4.9 kg/m^2^, p < 0.001 and waist: 95.8 ± 11.3 *vs*. 83.4 ± 12.4 cm, p < 0.001 than women. In men, the prevalences of overweight and obesity were 45.5% and 16.9%, respectively, higher than in women (28.3% and 14.3%, respectively, p < 0.001). In both genders, the prevalence of obesity increased with age (Table 1) and decreased with educational level: in women, from 23.9% in basic to 6.0% in university (p < 0.001); the corresponding values for men were 24.6% and 7.9% (p < 0.001). In women, the prevalence of obesity decreased from never smokers (17.8%) to former (12.9%) and current smokers (9.4%), whereas in men the prevalence of obesity was higher among former smokers (21.2%) than in never (14.6%) or current smokers (13.8%).

**Table 1:** prevalence of obesity and abdominal obesity by gender and age group.

Age groups (years)	[35–44]	[45–54]	[55–64]	[65–75]	Test
Women	N = 868	N = 915	N = 941	N = 525	
BMI status					
Normal	597 (68.8)	540 (59.0)	480 (51.0)	248 (47.2)	86.4
Overweight	188 (21.7)	244 (26.7)	300 (31.9)	186 (35.4)	***
Obese	83 (9.5)	131 (14.3)	161 (17.1)	91 (17.3)	
Abdominal obesity					
No	714 (82.3)	682 (74.5)	573 (60.8)	287 (54.5)	165.7
Yes	155 (17.7)	233 (25.5)	370 (39.2)	238 (45.3)	***
Men	N = 880	N = 846	N = 762	N = 449	
BMI status					
Normal	427 (48.5)	345 (40.9)	209 (27.4)	122 (27.2)	116.5
Overweight	353 (40.1)	375 (44.3)	383 (50.3)	225 (50.1)	***
Obese	100 (11.4)	125 (14.8)	170 (22.3)	102 (22.7)	
Abdominal obesity					
No	773 (87.8)	682 (80.6)	509 (66.8)	270 (60.1)	175.2
Yes	107 (12.2)	164 (19.4)	253 (33.2)	179 (39.9)	***

Results expressed as number of subjects (percentage). Statistical analysis by chi-square: ***, p < 0.001.

The prevalence of abdominal obesity was higher in women than in men (30.6% vs. 23.9%, p < 0.001), increased with age (Table 1) and decreased with educational level: in women, from 43.6% in those with basic education levels to 17.3% in those with university degrees (p < 0.001); the corresponding values were 24.8% and 15.6% for men (p < 0.001). In women, the prevalence of abdominal obesity decreased from never smokers (33.9%) to former (31.6%) and current smokers (23.3%), whereas, in men, it was higher among former (29.8%) than in never (19.4%) or current smokers (21.3%).

Multivariate analysis showed age and lack of leisure-time physical activity to be positively related and education to be negatively related with obesity and abdominal obesity in both genders, whereas a negative effect of smoking was found in women only (Table 2). Interestingly, the prevalence of abdominal obesity showed a steeper increase with age than BMI-derived obesity. In men, a steeper decrease in the prevalence of BMI-derived obesity was found with educational level (Table 2).

**Table 2:** factors related to obesity and abdominal obesity, by gender.

	Women (n = 3,249)		Men (n = 2,937)	
	Obesity	Abdominal obesity	Obesity	Abdominal obesity

Age groups				
[35 – 44]	1 (ref.)	1 (ref.)	1 (ref.)	1 (ref.)
[45 – 54]	1.56 [1.15 – 2.11]	1.55 [1.23 – 1.96]	1.23 [0.92 – 1.64]	1.61 [1.23 – 2.11]
[55 – 64]	1.75 [1.31 – 2.35]	2.79 [2.23 – 3.49]	2.03 [1.54 – 2.68]	3.25 [2.52 – 4.21]
[65 – 75]	1.50 [1.07 – 2.09]	3.19 [2.48 – 4.12]	1.91 [1.40 – 2.62]	4.27 [3.21 – 5.67]
Test for trend	6.3 *	101.7 ***	24.0 ***	125.5 ***
Educational level				
Basic	1 (ref.)	1 (ref.)	1 (ref.)	1 (ref.)
Apprenticeship	0.64 [0.50 – 0.81]	0.69 [0.57 – 0.84]	0.75 [0.58 – 0.96]	1.18 [0.92 – 1.52]
High school	0.41 [0.30 – 0.55]	0.48 [0.38 – 0.60]	0.55 [0.40 – 0.74]	1.07 [0.81 – 1.42]
University	0.27 [0.18 – 0.40]	0.38 [0.29 – 0.50]	0.30 [0.21 – 0.43]	0.70 [0.51 – 0.96]
Test for trend	45.2 ***	52.5 ***	47.5 ***	5.58 *
Smoking status				
Never	1 (ref.)	1 (ref.)	1 (ref.)	1 (ref.)
Former	0.74 [0.58 – 0.95]	0.99 [0.82 – 1.19]	1.36 [1.08 – 1.73]	1.42 [1.15 – 1.76]
Current	0.46 [0.35 – 0.61]	0.63 [0.51 – 0.77]	0.79 [0.60 – 1.04]	0.99 [0.78 – 1.26]
Test for trend	29.3 ***	20.1 ***	2.75 ^NS^	0.01 ^NS^
Leisure time PA				
≥1/week	1 (ref.)	1 (ref.)	1 (ref.)	1 (ref.)
None	2.06 [1.67 – 2.53]	1.74 [1.48 – 2.05]	1.54 [1.25 – 1.89]	1.77 [1.47 – 2.13]

Results are expressed as Odds ratio and [95% confidence interval]. PA: physical activity. Statistical analysis by logistic regression: ^NS^, not significant; *, p < 0.05; ***, p < 0.001.

## Discussion

The prevalence of obesity in the population of Lausanne was lower than in neighboring countries [12,13] but higher than previous estimations for Switzerland [14,15], probably due to differences in age. The increase in obesity levels with age is of concern, as it has been shown that obese elderly are more likely to present with major chronic health conditions and poor general health [14].

Increased waist has been shown to be related to cardiovascular risk factor levels [16] and all-cause mortality [17], and to be a better risk indicator than BMI [2,4], although this latter statement has been challenged [18]. In this study, the prevalence of abdominal obesity was higher than the prevalence of obesity defined by BMI. Those findings indicate that a significant part of the population not classified as obese based on BMI levels might actually be at higher health risk due to increased waist. Further, and similar to other studies [12,13,19], the differences between BMI- and waist-defined obesity levels were higher in women than in men ; whereas the prevalence of obesity tended to plateau with age, abdominal obesity increased dramatically between decades 3 and 4. These findings indicate that the prevalence of obesity might be considerably underestimated among middle-aged and elderly women, which precludes them from benefiting from preventive measures. As waist is a simple and inexpensive measurement significantly related to cardiovascular risk factor levels [16] and all-cause mortality [17], it could be preferred to BMI or to body fat measurements to screen subjects at risk, but it is difficult to assess and may have a wide inter-observer variation. Another potential explanation for the considerable difference in BMI- or waist-derived obesity prevalences between genders could be related to the fact that BMI-related obesity is based on a single cut-off for both genders, whereas gender-specific cut-offs were used for waist-derived obesity. Indeed, it has been suggested that a single BMI cut-off might not be adequate to define obesity, and that ethnic [20,21], gender [21], age [22] and even socio-economic [23] specific levels might be preferable.

The decrease in obesity prevalence with increasing educational level is in agreement with the literature [24-26] and might partly be related to differences in dietary intake, subjects with a lower socioeconomic level being more prone to buy cheaper, energy-dense foods [27,28]. The decrease in obesity prevalence with smoking has also been repeatedly reported [25], and has been related to a higher resting metabolic rate [29] and to a different dietary intake among smokers relative to non-smokers [30]. The differential effects of smoking on obesity levels between men and women were unexpected, and might be related to the fact that heavy smoking increases BMI [31] or that women smoke, in part, to control weight, but further studies are needed to better assess this point.

The present study suffers from some limitations. First, the participation rate was rather low (41%), which might limit the generalizability of our findings. However, low participation is typical of surveys in Western countries and is comparable with the MONICA surveys conducted in Switzerland and in other countries [32]. The magnitude of the non-participation bias is not proportional to the percentage of non-participants [33] and a study on representativeness observed that people with risky behaviours participated in the same proportions as people without risk factors [34]. Second, only subjects of Caucasian origin were included in this study, and our findings therefore do not apply to other ethnic groups. It may be argued thatthe genetic mix of Caucasians in Lausanne is not representative of the whole country. However, a considerable proportion of the Lausanne population is non-Swiss or comes from other cantons, including individuals of Italian or Germanic origin: in 2006, out of the 128,231 Lausanne inhabitants, 49,330 (38%) were non-Swiss, 38,513 (30%) came from other cantons, and only 40,388 subjects (32%) were actually from the canton of Vaud [35]. We thus believe that the genetic mix of the CoLaus sample is relatively large and that the results may be extrapolated with reasonable confidence to the Swiss population.

## Conclusion

The prevalence of abdominal obesity is higher than BMI-derived obesity in the Lausanne population. Women present with more abdominal obesity than men. The effect of smoking on obesity levels appears to differ between genders.

## Competing interests

Vincent Mooser is a full-time employee of GlaxoSmithKline.

## Authors' contributions

PMV and MB made the statistical analysis and wrote part of the article. FP, VM, GW and PV contributed to the statistical analysis design, wrote part of the article and made major corrections.

## Pre-publication history

The pre-publication history for this paper can be accessed here:


